# Limb Salvage Using Polytetrafluoroethylene Prosthetic Bypass in Femoral Atherosclerosis: A Case Report and Review of Literature

**DOI:** 10.7759/cureus.38779

**Published:** 2023-05-09

**Authors:** Aditya Sharma, Rajeshwar Yadav, Swati Pathak

**Affiliations:** 1 Department of General Surgery, Institute of Medical Sciences, Banaras Hindu University, Varanasi, IND; 2 Department of Cardiothoracic and Vascular Surgery, Institute of Medical Sciences, Banaras Hindu University, Varanasi, IND

**Keywords:** saphenous venous graft ( svg), atherosclerosis (as), common femoral artery stenosis, polytetrafluoroethylene(ptfe), femoropopliteal bypass

## Abstract

The choice of a vascular graft in patients with femoral atherosclerosis has always been a matter of debate. But when a deep review of the literature is done, the autogenous graft using the saphenous vein is considered the most reliable graft material for the vessels involved below the level of the inguinal ligament. In recent years, there have been published studies comparing vascular versus prosthetic grafts. We report a case covering a similar domain where femoropopliteal bypass was done using a polytetrafluoroethylene (PTFE) prosthetic graft and the outcome after the surgical procedure.

## Introduction

Bypass procedures are considered a procedure for salvaging a limb in patients suffering from atherosclerotic changes in vessels that can produce peripheral arterial ischemia [1]. It is very important to achieve revascularization in such patients, which can be accomplished with the placement of a bypass graft [2]. The grafts used in bypass surgical procedures for atherosclerotic vessels have evolved over time.

The choice of graft, whether venous or prosthetic, depends on several factors, which vary from patient to patient and from surgeon to surgeon. Patient factors include the unavailability of an adequate saphenous venous graft and financial conditions, while a surgeon's factors include preference and expertise in dealing with such grafts [3]. We report a similar case where there was a dramatic improvement in extensive symptoms of atherosclerosis after the placement of a prosthetic polytetrafluoroethylene (PTFE) graft.

## Case presentation

A 53-year-old non-diabetic man, smoker, and hypertensive presented to the cardiothoracic and vascular surgery outpatient department with the chief complaints of intermittent claudication in his right leg for six months. On examination, peripheral pulses like popliteal, posterior tibial, and dorsalis pedis were non-palpable in the right leg, and the limb was cold with overlying skin changes and capillary refill time of more than three seconds, and brittle nails in comparison to the contralateral limb. In the left lower limb, the peripheral pulses were palpable, capillary refill time was less than three seconds, and there were no overlying skin changes with the normal texture of nails.

The patient's pulse was 80 beats per minute, regular in rhythm, and the blood pressure was 120/70 mm Hg on antihypertensive medications. His pulses were palpable in both upper limbs. Motor and sensory functions were intact in both lower limbs. A duplex study was obtained in assessing the bilateral lower limbs, which revealed 100% occlusion of the femoropopliteal segment in the right lower limb and normal venous systems in both lower limbs. The ECG and 2D echo were also done and the findings were within normal limits.

**Figure 1:**
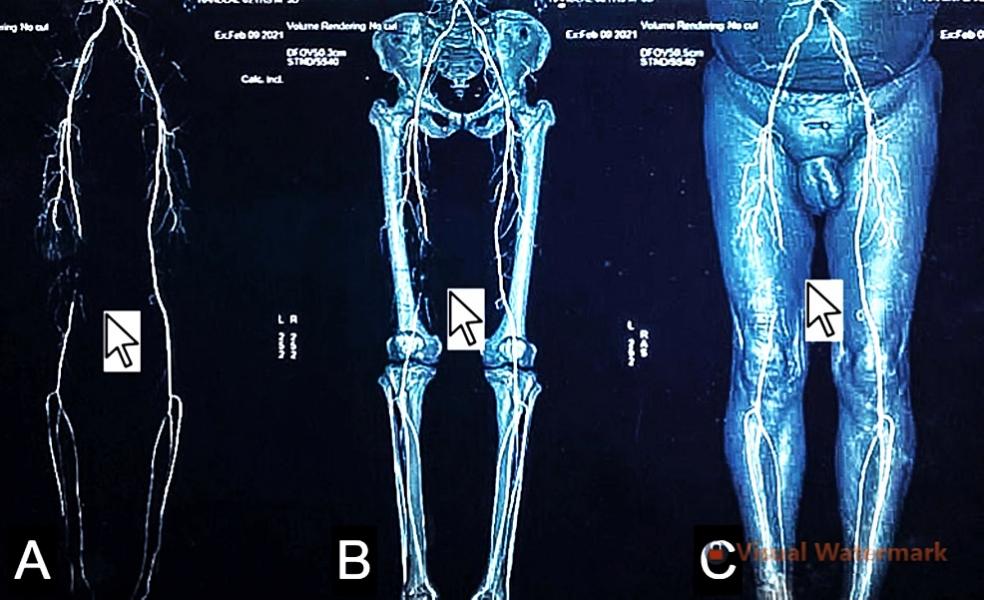
On computed tomography angiography, a complete filling defect of the superficial femoral artery was noted at the level of the mid-thigh with distal recanalization just above the level of the knee, features suggestive of thrombosis, as seen in (A) the reconstruction of arteries without bony and soft tissues, (B) the arterial system in relation to the bones, and (C) the arterial system with bone and soft tissue.

The computed tomography angiography findings in the left lower limb were within normal limits, while in the right lower limb, there revealed a complete filling defect of the superficial femoral artery at the level of the mid-thigh with distal recanalization just above the level of the knee, features suggestive of thrombosis. The right popliteal, anterior tibial and posterior tibial arteries show normal contrast opacification, as shown in Figures 1A-1C.

After subjecting the patient to all these investigations, a femoropopliteal bypass for the right lower extremity was recommended to the patient using a PTFE graft. The patient was shifted to the operation theater and placed under epidural anesthesia in a supine position. Initially, urethral catheterization was done. Painting and draping were done from the umbilicus down to the mid-calf region. A skin incision was made in the right mid-thigh area along the course of the superficial femoral artery, and arterial vessels were isolated. An incision was extended along the distal femoral artery up to the adductor canal to expose the popliteal artery above the knee.

Systemic heparinization with a bolus of 5,000 units of unfractionated heparin was achieved. The proximal and distal controls were established. The proximal right superficial femoral artery and right distal popliteal artery bypass graft was placed using 6-0 polypropylene sutures and a 6-mm PTFE graft as venous graft was insufficient in this patient due to varying anatomy of the venous vasculature, thereby establishing a femoropopliteal bypass graft from the right distal superficial femoral artery to the right popliteal artery, just above the knee.

It was ensured that there would be no twisting or kinking of the graft. An intravenous sodium bicarbonate injection was given just before releasing the arterial control clamps. Good flow in the graft was ensured before closing the wound. The wound was closed with a drain kept in situ, which was removed on postoperative day 2. All peripheral pulses demonstrated strong pulsation, with oxygen saturation of 100% in both lower limbs. The patient was kept on intravenous heparin with dextran infusion (low molecular weight heparin, 40,000 units) for three days.

**Figure 2:**
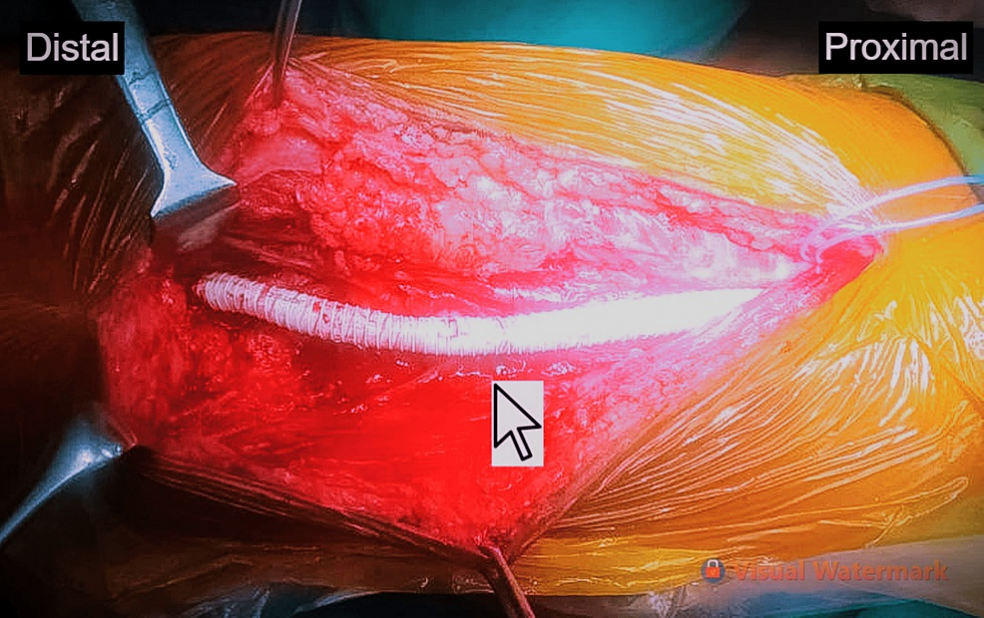
A 6-mm polytetrafluoroethylene (PTFE) graft, establishing a femoropopliteal bypass graft from the right distal superficial femoral artery to the popliteal artery, above the level of the knee.

Thereafter, the patient was switched to oral antiplatelet, statins and anticoagulant therapy. His postoperative recovery was uneventful. The claudication improved and disappeared 10 days postoperatively. The patient was discharged on the 14th postoperative day with the advice of regular dressing changes, continue the prescribed medications, and to be seen at a follow-up visit. The placement of the prosthetic graft is shown in Figure 2.

## Discussion

In this case, we have convincingly learned that in patients where the vascular grafts, such as great saphenous venous grafts, are not sufficient to carry out bypass procedures because of the narrow veins and varying vasculature, prosthetic grafts like PTFE may be employed to carry out limb salvage bypass surgeries in order to restore vascularization [4].

The expected postoperative complications can include graft thrombosis, bleeding, and local site wound infection. The most important marker regarding the success of such bypass surgery is the ultimate patency of the graft as demonstrated with adequate vascular flow through the graft [5].

And as far as patency is concerned, such grafts used in femoropopliteal bypass surgeries have shown the best outcomes, with around 90% of these grafts remaining patent for years, but the same cannot be taken as the principle for symptomatic control as most of the patients might complain of intermittent symptoms postoperatively [6]. Femoropopliteal bypass surgery is one of the gold standards employed in the surgical management of occlusive vascular ischemic disease and if performed correctly may result in a successful outcome, as seen in the present case.

## Conclusions

We are reporting this case to elicit the successful management of a patient presenting with occlusive vascular ischemic disease and how timely intervention and the use of a prosthetic PTFE graft was utilized because the greater saphenous vein as a graft was not acceptable in this patient. This case may sensitize the surgeons performing the vascular bypass surgery using conventional methods to the fact that, apart from the vascular grafts, these prosthetic grafts also have outstanding outcomes when revascularizing a lower limb and demonstrate rapid improvement post-operatively, as observed in our patient.
